# Transcriptomic and Metabolomic Insights into Key Genes Involved in Kinsenoside Biosynthesis in *Anoectochilus roxburghii*

**DOI:** 10.3390/plants14050688

**Published:** 2025-02-24

**Authors:** Peiyu Wang, Peipei Yan, Zunwen Li, Jinlan Jiang, Yuling Lin, Wei Ye

**Affiliations:** 1 Sanming Academy of Agricultural Sciences, Shaxian 365051, China; 17268144914@163.com (P.W.);; 2 The Key Laboratory of Crop Genetic Improvement and Innovative Utilization in Fujian Province (Mountain Area), Shaxian 365051, China; 3 Institute of Horticultural Biotechnolngy Fujian Aoriculture and Forestry University, Fuzhou 350002, China

**Keywords:** *Anoectochilus roxburghii*, kinsenoside, high-performance liquid chromatography, transcriptome, metabolite

## Abstract

As the main active ingredient in *Anoectochilus roxburghii,* kinsenoside has important health and medical effects including hepatoprotective, anti-oxidant, and bacteriostasis, among others. In recent years, with the limited application of high-throughput technology to *A. roxburghii*, there has been no research on the key regulatory genes involved in the synthesis of kinsenoside. Therefore, we examined three species of *A. roxburghii* that are widely planted in mainland China and Taiwan Province, *A. roxburghii* cultivar ‘Jian ye’, *Anoectochilus formosanus*, and *Anoectochilus burmannicus*, determining the content of kinsenoside, performing transcriptomic and metabolomic sequencing, identifying UDP glycosyltransferases, and screening for UDP glycosyltransferases that may be involved in kinsenoside synthesis. The results showed that among the three species of *A. roxburghii*, the content of kinsenoside in *A. roxburghii* cv. ‘Jian ye’ was the highest. Transcriptome and metabolome data showed that *A. roxburghii* cv. ‘Jian ye’ and the two other species of *A. roxburghii* have 3702 and 5369 differentially expressed genes and 69 and 120 differentially accumulated metabolites, respectively. Meanwhile, differentially expressed genes and differentially accumulated metabolites are enriched in the glucose metabolism and hormone pathways. We also treated the *A. roxburghii* samples with exogenous auxin and characterized the related genes. In *A. roxburghii*, we identified 73 members of the UDP glycosyltransferase family. Through phylogenetic tree, transcriptome data expression profile, and qPCR analyses, we screened for members that may be involved in the synthesis of kinsenoside. In summary, the results of this study provide insights for breeding high-kinsenoside-content and high-intron varieties of *A. roxburghii*.

## 1. Introduction

*Anoectochilus roxburghii* (Wall.) Lindl. is a precious perennial herb of the *Orchidaceae* family, belonging to the genus *Anoectochilus. A. roxburghii* is widely distributed in the Himalayas and tropical and subtropical areas from Southeast Asia to Hawaii. The main areas of *A. roxburghii* production in China are Fujian Province, Taiwan Province, Yunnan Province, and Guangdong Province [1]. The main bioactive components in *A. roxburghii* are polysaccharides, flavonoids, glycosides, amino acids, etc. Among them, kinsenoside is one of the most important active ingredients. Pharmacological studies have shown that kinsenoside has significant biological activities, such as reducing blood lipids, protecting the liver, lowering blood sugar, and anti-inflammatory and anti-oxidant effects [2,3]. However, even though the content of kinsenoside in *A. roxburghii* is high, it is still very expensive to extract kinsenoside using traditional methods. Therefore, understanding the genes involved in kinsenoside biosynthesis in plants is of great significance for breeding and creating high-kinsenoside germplasms.

The transcriptome is usually divided into broad and narrow categories. The broad transcriptome refers to the sum of all transcription products in living cells, including non-coding RNA (microRNA, tRNA, snRNA, etc.) and protein-coding RNA (mRNA). The narrowly defined transcriptome refers to its mRNAs [4]. As a widely used high-throughput sequencing method, transcriptomics is used extensively in plant research due to its low cost and powerful functionality. Although genomics research on *A. roxburghii* started relatively late, and there are no reports on its genomic information as of yet, transcriptomics technology is gradually being used to study the functional genes and genetic mechanisms of *A. roxburghii*. Liu et al. used transcriptome sequencing to identify key genes related to heat stress in *A. roxburghii* [5], and Cui et al. used high-throughput sequencing technology to investigate dynamic changes in flavonoids in *A. roxburghii* under heat stress [6]. Huang et al. discovered an albino *A. roxburghii* and found through biochemical and transcriptomic techniques that its leaves had underdeveloped chloroplasts, low pigment content, and downregulated related genes and metabolites [7]. Zhang et al. found that genes related to flavonoid synthesis were highly expressed in *A. roxburghii*, which was symbiotic with the mycorrhizal fungus *Ceratobasidium* sp. AR2, promoting the accumulation of flavonoids [8,9]. In summary, high-throughput transcriptome sequencing has been carried out gradually in *A. roxburghii*; however, it has not yet been used to analyze the key genes involved in kinsenoside biosynthesis.

UDP glycosyltransferase can catalyze sugar molecules as donors of sugar groups, glycating a large number of secondary metabolites such as plant hormones, flavonoids, and terpenes [10,11]. The structure of kinsenoside shows that it is composed of six-carbon glucose and 3-hydroxy-γ-butyrolactone (3HBL). Previous studies have suggested that there must be a pathway in nature for the synthesis of kinsenoside through the glycosylation of 3HBL [12,13]. Therefore, the UDP glycosyltransferase gene family may play an important role in the synthesis of kinsenoside.

Taking three species of *A. roxburghii* as materials (*A. roxburghii* cultivar ‘Jian ye’, *A. formosanus,* and *A. burmannicus*), we explored the potential role of UDP glycosyltransferase in the biosynthesis of kinsenoside. Firstly, we conducted a comprehensive transcriptomic and metabolomic analysis of three *A. roxburghii* materials and quantified their kinsenoside content using high-performance liquid chromatography. Through a correlation analysis of the transcriptome and metabolome, it was found that differentially expressed genes (DEGs) and differentially accumulated metabolites were enriched in the glucose metabolism and auxin pathways. We also identified UDP glycosyltransferase members in the transcriptome of *A. roxburghii* and performed phylogenetic analysis with all members of *Arabidopsis*. In addition, highly expressed members in of *A. roxburghii* were identified through transcriptome data and subjected to real-time fluorescence quantitative PCR (RT-qPCR) analysis, and the patterns of their response to auxin were analyzed. In summary, these results provide a molecular biology basis for the development of functional compounds and germplasm innovation in *A. roxburghii*.

## 2. Materials and Methods

### 2.1. Test Materials

Three samples of *A. roxburghii* were selected from each species: *A. roxburghii* cv. ‘Jian ye’ (resource conservation number A040), *Anoectochilus formosanus* (resource conservation number A009), and *Anoectochilus burmannicus* (resource conservation number A043). All materials were stored in the form of tissue-cultured seedlings at the Institute of Medicinal Plants, Sanming Academy of Agricultural Sciences, Fujian Province. During sampling, the whole plant was used as the sample of *A. roxburghii*. Three biological replicates were collected for each sample and rapidly frozen in liquid nitrogen. The samples were then stored in a −80 °C freezer for later use.

### 2.2. Kinsenoside Content Detection

This study selected tissue-cultured *A. roxburghii* seedlings that had entered the strong seedling stage after 6 months of cultivation and had consistent growth. Fresh or dry samples were crushed and sieved through a No. 4 sieve. Then, 0.150 g of the sample was accurately weighed and placed in a stoppered triangular flask. To this, 50.0 mL of 40% methanol solution was added. The flask was then sealed tightly, weighed, sonicated for 10 min, cooled, and weighed again. The weight loss was compensated with a 40% methanol solution; the sample was then shaken well and filtered through a 0.45 mm filter membrane to obtain the sample solution. The analytical instruments consisted of an Agilent 1260 high-performance liquid chromatography (HPLC) system and an Alltech 3300 evaporative light-scattering detector (ELSD; GRACE, Deerfield, MA, USA). The chromatographic column was an amino column (4.6 mm × 250 mm, 5 μm; mobile phase: acetonitrile–water (85:15) (volume ratio); flow rate: 1.0 min/mL; atomization chamber temperature: 70 °C; injection volume: 10 μL; N2 flow rate: 1.5 L/min; gain value: 8; injection volume: 10 μL). We performed high-performance liquid chromatography separation and detection and a quantitative comparison with standard substances. The content of kinsenoside in the sample was calculated using the formula X = C × V × 100/m × 1000, and the calculation result was kept to 3 digits. In the formula, X—the content of kinsenoside in the sample, measured in g/100 g; c—the concentration of kinsenoside in the sample solution, in units of mg/mL; V—the volume of the sample solution, in units of mL; m—the mass of the sample, calculated on a dry basis, in units of g; 100—g/g is converted as a coefficient of g/100 g; 1000—mg is converted as a coefficient of g. Finally, the content of kinsenoside obtained is analyzed using SPSS 22.0 software, and the significance of their differences is calculated using the least significant difference (LSD) method, *p* < 0.05.

### 2.3. Transcriptome Data Assembly of A. roxburghii

Three species of tissue-cultured *A. roxburghii* seedlings were rapidly frozen in liquid nitrogen and subjected to transcriptome library construction and paired-end sequencing at Baimaike Biotechnology Company. Nine sets of transcriptome data from three species of *A. roxburghii* were first filtered using fastp (v0.20.1) software to remove low-quality data [14]. Subsequently, Trinity (v2.14.0) software was used to break the sequencing reads into shorter fragments and extend them into longer contigs [15]. At the same time, the coincidence between fragments was used to obtain the fragment set, and finally, the De Bruijn map method and sequencing read information were used to identify the transcript sequences in each fragment set [16].

### 2.4. Transcript Annotation and Analysis of Differentially Expressed Genes of A. roxburghii

The sequence of the coding region and the corresponding amino acid sequence of the transcripts were predicted using TransDecoder (v5.0.0) (https://github.com/TransDecoder/TransDecoder, accessed on 19 October 2023) software. The filtered transcriptome data were aligned to the transcripts using Bowtie software (v2.3.4.1) [17]. Subsequently, the gene expression levels were calculated using RSEM (v1.2.19) software [18]. Finally, the DND was analyzed using DESeq2 (v1.30.1) software, with Fold Change 2 and FDR < 0.01 as screening criteria [19].

### 2.5. Metabolite Extraction, Identification, and Quantification

To understand the dynamic changes in metabolites in different species of *A. roxburghii*, extensive targeted metabolomics was used to analyze samples from the three species of *A. roxburghii* at Qingdao Baimaike Biotechnology Co., Ltd. (Qingdao, Shandong, China), with a set of three replicates for each sample. First, the freeze-dried samples of *A. roxburghii* plants were ground thoroughly, and 50 mg of the resulting powder was added to 1 mL of 70% methanol and extracted overnight at 4 °C. Subsequently, the supernatant was collected via centrifugation at 10,000× *g* rpm, and the types and contents of substances in the supernatant were identified using the UHPLC-QE Orbitrap platform [20]. Firstly, we performed principal component analysis (PCA) on the obtained metabolome data to detect its repeatability. Subsequently, a variable importance projection was obtained based on OPLS-DA model analysis results (Variable Importance in Projection, VIP). One of the screening criteria for differential metabolites is VIP > 1. In addition, differential metabolites were screened for metabolite content at different stages using another criterion: |log2Fold Change| > 1. We annotated and enriched all metabolites and genes by the Kyoto Encyclopedia of Genes and Genomes (KEGG) compound database (http://www.kegg.jp/kegg/compound/, accessed on 19 October 2023) and the KEGG pathway database (http://www.kegg.jp/kegg/pathway.html, accessed on 19 October 2023).

### 2.6. Weighted Gene Co-Expression Analysis

To obtain the relevant genes involved in kinsenoside synthesis in *A. roxburghii*, weighted gene co-expression network analysis (WGCNA) was used for gene co-expression network analysis and gene module identification [21]. This study normalized the fragments per kilobase of exon model per million mapped fragments (FPKM) values of all genes in *A. roxburghii* and used the kinsenoside content as phenotype data for comprehensive analysis.

### 2.7. UDP-Glycosyltransferase Gene Family Identification and Phylogenetic Tree

The entire protein data set of *Arabidopsis thaliana* was downloaded from TAIR (The Arabidopsis Information Resource) database (v 11). Subsequently, we utilized InterProScan (v5.59-91.0) software to annotate the domain of all protein sequences for both *A. thaliana* and *A. roxburghii* [22]. We opted to screen all gene members using the Pfam database (PF08449) and the PANTHER database (PTHR10778), defining the intersection of genes identified by both databases as members of the UDP glycosyltransferase gene family. Firstly, we used Muscle (v3.8.31) to a perform multiple sequence alignment of UDP glycosyltransferase protein sequences in *Arabidopsis* and *A. roxburghii* [23]. Subsequently, phyutility software (v2.2.6) (https://github.com/blackrim/phyutility, accessed on 19 October 2023) was used to remove gaps from all sequences, using prottest software (v3.3) (https://github.com/ddarriba/prottest3, accessed on 19 October 2023) to search for the most reasonable evolutionary tree to construct a model. Finally, Iqtree (v2.3.5) software was used to construct phylogenetic trees [24].

### 2.8. Quantitative Analysis

Total RNA was extracted from the three species of *A. roxburghii* using the TRIzol^TM^ RNA extraction kit (Thermo Fisher, 15596026, Waltham, MA, USA), and 3 biological replicates were established. The RNA concentration of each sample was determined using a NanoDrop 2000 (Thermo Scientific, Waltham, MA, USA), and the integrity of the RNA was determined using gel electrophoresis. The total RNA was subsequently reverse-transcribed to quantify the desired cDNA using the PrimeScript^TM^ RT reagent reverse transcription kit (TaKaRa, Tokyo, Japan), according to the instructions. RT-qPCR was performed using an ABI QuantStudio 3-sequence detection system (Applied Biosystems, Waltham, MA, USA) and the SYBR Premix Ex Taq Kit (TaKaRa, Tokyo, Japan), with the following reaction conditions: 95 °C for 3 min, 40 cycles of 95 °C for 15 s, 56 °C for 30 s, and 72 °C for 20 s. The reference gene Actin was used for normalization; the expression data were calculated using the 2^−∆∆Ct^ formula, and the difference was calculated using SPSS 22.0 software (LSD method, *p* < 0.05). The primers used for quantification are listed in the Supplementary Materials, Table S1.

## 3. Results and Analysis

### 3.1. Determination of Kinsenoside Content and Evaluation of Sequencing Data Quality for Three Species of A. roxburghii

**Figure 1:**
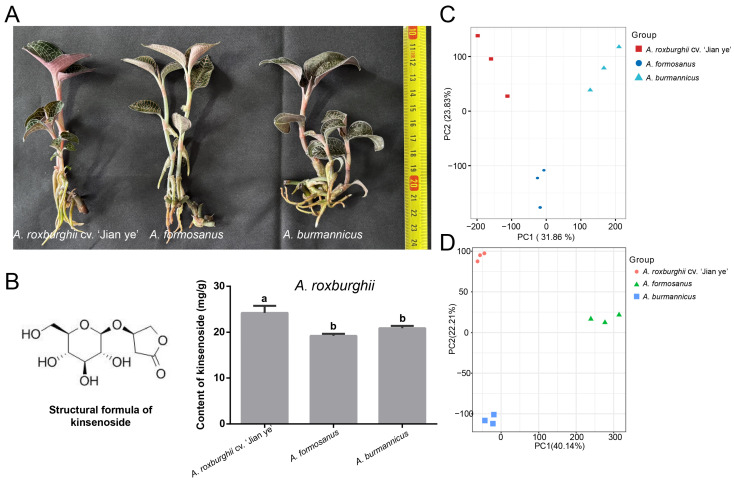
Determination of kinsenoside content and transcriptome data clustering of *A. roxburghii.* (**A**) Three species of tissue-cultured *A. roxburghii* seedlings. (**B**) Kinsenoside molecular structure and content determination. Lowercase letters indicate significant differences. (**C**) PCA of transcriptome data. (**D**) PCA of metabolome data.

In this study, we took three species of *A. roxburghii* (‘Jian ye’, *A. formosanus,* and *A. burmannicus*) that are widely planted in the Chinese mainland and Taiwan Province and isolated them from their mother plants for stem-section tissue culture. After 180 days of culture, whole-plant samples demonstrating the same growth trend were taken (Figure 1A). Furthermore, the content of kinsenoside was measured by the high-performance liquid chromatography method. For each sample of *A. roxburghii*, the entire plant was frozen using liquid nitrogen and ground thoroughly. A standard curve was drawn based on the kinsenoside produced using a high-performance liquid chromatography (HPLC-ELSD) method, and the content of kinsenoside was measured. The results show that the kinsenoside content in the *A. roxburghii* cv. ‘Jian ye’ variety was significantly higher than in the other two species of *A. roxburghii* (*p* < 0.05). (Figure 1B). To understand the genes and key metabolites that may be involved in kinsenoside synthesis in *A. roxburghii*, we conducted transcriptomic and metabolomic analyses. PCA shows that the gene expression levels and contents of the three species can be separated effectively, and the biological replicates can be successfully clustered as well. Both principal component 1 (PC1) and principal component 2 (PC2) exhibit high capture rates (Figure 1C,D).

### 3.2. Transcriptome and Metabolome Data Analysis for the Three Species of A. roxburghii

Transcriptome sequencing, the most widely used and effective high-throughput sequencing method, is typically used to study the transcriptional regulation of genes. Transcriptome sequencing was performed on samples consisting of the entire plants from three species of *A. roxburghii*, and three replicates of the samples from each species were selected. The sequencing yielded 6.10–8.23 Gb of raw data, with Qphred 30 filtering by fastp software (Q30; the probability of correct sequencing for one base is 99.99%) of all data being greater than 92.38% (Supplementary Materials, Table S2). The results revealed a high quality of transcriptome data for sequencing, indicating that it could be used for further analysis. To understand the key genes regulating kinsenoside synthesis, we compared the combinations of *A. roxburghii* cv. ‘Jian ye’ vs. *A. formosanus* and *A. roxburghii* cv. ‘Jian ye’ vs. *A. burmannicus*. There were 3702 DEGs between *A. roxburghii* cv. ‘Jian ye’ and *A. formosanus*, of which 1683 genes were upregulated and 2019 genes were downregulated (Figure S2A,C). However, there were 5369 DEGs between *A. roxburghii* cv. ‘Jian ye’ and *A. burmannicus*, of which 2673 genes were upregulated and 2696 genes were downregulated (Figure S2B,C). In addition, 1782 shared DEGs were included in the two combinations (Figure S2D). Therefore, we hypothesized that these 1782 DEGs should contain key genes regulating kinsenoside synthesis. Furthermore, according to the OPLS-DA method, we have confirmed the effective model for our differential metabolite analysis (Figure S3). Our metabolomic analysis revealed 69 differentially accumulated metabolites between *A. roxburghii* cv. ‘Jian ye’ and *A. formosanus*, with 65 accumulating significantly and 4 decreasing (Figure S4A,C). There were 120 differentially accumulated metabolites between *A. roxburghii* cv. ‘Jian ye’ and *A. burmannicus*, of which 78 accumulated significantly and 42 decreased in accumulation (Figure S4B,C). Meanwhile, there were 36 metabolites shared among them (Figure S4D).

**Figure 2:**
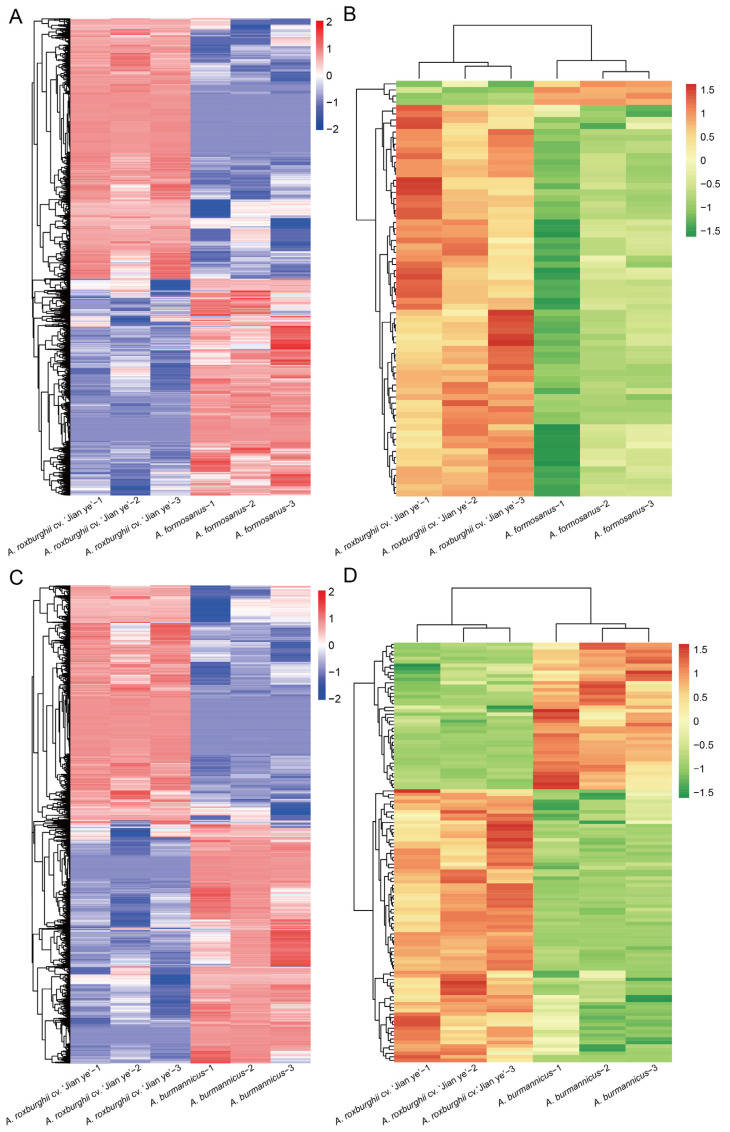
Cluster analysis of DEGs and differentially accumulated metabolites in different varieties of *A. roxburghii*. (**A**,**B**) Clustering of DEGs and differentially accumulated metabolites in *A. roxburghii* cv. ‘Jian ye’ and *A. formosanus*. The colors in clustering represent the expression levels of genes in the sample log10 (FPKM + 0.000001). (**C**,**D**) Clustering of DEGs and differentially accumulated metabolites in *A. roxburghii* cv. ‘Jian ye’ and *A. burmannicus*. The colors in the clustering represent the standardized Z-score values of metabolites after hierarchical clustering of metabolite content.

**Figure 3:**
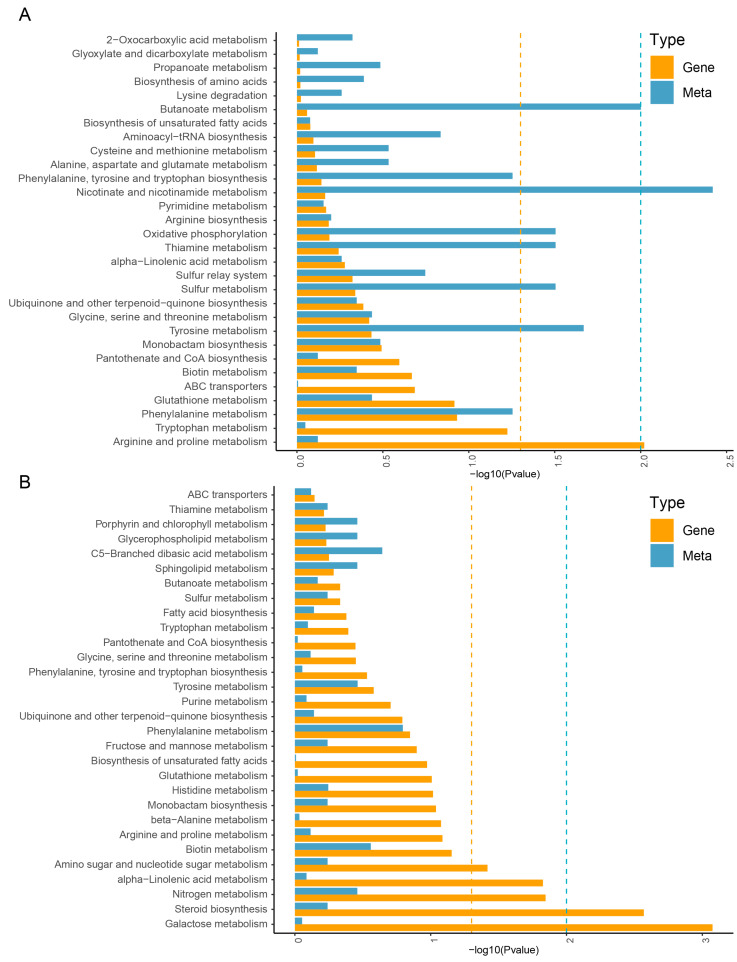
KEGG enrichment analysis results of DEGs and differentially accumulated metabolite. (**A**) Combined KEGG enrichment analysis of DEGs and differentially accumulated metabolites in *A. roxburghii* cv. ‘Jian ye’ and *A. formosanus*. (**B**) Combined KEGG enrichment analysis of DEGs and differentially accumulated metabolites in *A. roxburghii* cv. ‘Jian ye’ and *A. burmannicus*.

In addition, a further clustering analysis was conducted on DEGs and differentially accumulated metabolites among the three samples (Figure 2A–D). The results showed that the expression patterns of three replicates in the same species of *A. roxburghii* were similar, while there were significant differences in gene expression among different species. Therefore, in order to understand the similarities and differences in the functions of DEGs and differentially accumulated metabolites, we further conducted a KEGG enrichment analysis on the correlation between DEGs and differentially accumulated metabolites and selected the top 30 pathways for visualization (Figure 3A,B). The results showed that the KEGG pathways enriched with DEGs and metabolites between *A. roxburghii* cv. ‘Jian ye’ and *A. formosanus* were similar in height to those between *A. roxburghii* cv. ‘Jian ye’ and *A. burmannicus*. In addition, 17 out of the top 30 KEGG pathways in the two comparison groups were the same. These mainly focused on the metabolism of vitamins, cofactors, amino acids, lipids, and other substances. This indicates that genetic background differences between species may affect their metabolic characteristics, which in turn may lead to different species exhibiting different traits in terms of physiological functions, medicinal value, or adaptability. *A. roxburghii* cv. ‘Jian ye’ shows unique gene expression patterns or metabolite accumulation on these metabolic pathways, which may result in specific biological characteristics or application value.

### 3.3. Construction of Kinsenoside Synthesis Regulatory Network in A. roxburghii

To investigate the key genes involved in kinsenoside synthesis in *A. roxburghii*, we conducted an iterative analysis using the expression level data of all genes and the content of kinsenoside. We use WGCNA technology to screen gene modules that were highly correlated with kinsenoside, using the kinsenoside content as phenotypic data. The results showed that all genes in *A. roxburghii* are divided into nine modules (Figure S5). Among the nine modules, the green-yellow and black modules are highly positively correlated with the content of kinsenoside, with Pearson correlation values of 0.78 and 0.88, respectively. In addition, we also discovered a gene module that is significantly negatively correlated with the content of kinsenoside, MEIdningblue, with a Pearson correlation value of 0.65. These results suggest that the synthesis of kinsenoside may be regulated via a gene network (Figure S6).

**Figure 4:**
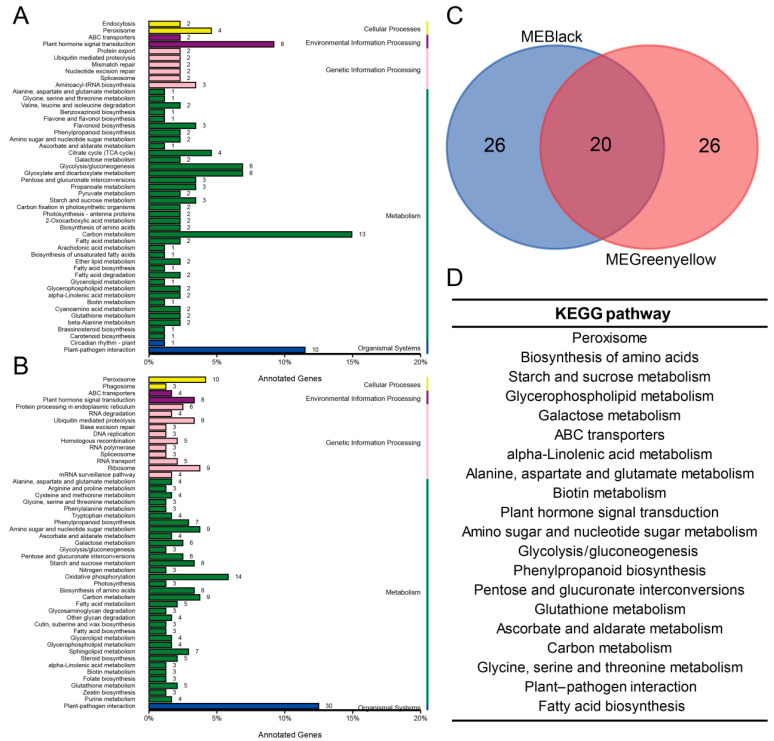
KEGG analysis of gene modules significantly correlated with kinsenoside. (**A**,**B**) The KEGG pathway of genes enriched in the green-yellow module and black gene modules. (**C**) Venn diagram analysis of the two modules’ KEGG pathways. (**D**) The KEGG pathways shared by both modules.

The green-yellow module contains a total of 748 genes. Further KEGG enrichment analysis revealed that they are mainly enriched in plant sugar metabolism, amino acid metabolism, signal transduction and regulation, biosynthesis, and interpretation, as well as cellular processes and molecular mechanisms. The black module contains a total of 1561 genes, and its KEGG pathway result was highly similar to that of the green-yellow module (Figure 4A,B). Therefore, we plotted a Venn diagram of the KEGG pathway enrichment results for the two modules. The results showed that the black module had 26 unique KEGG pathways, while the green-yellow module had 26 specific pathways. The two modules had 20 KEGG pathways in common (Figure 4C). Among the 20 KEGG metabolic pathways shared between the two modules, we found a large number of KEGG pathways related to sugar metabolism and hormone metabolism that were common to the two modules (Figure 4D). The above results indicate that glucose metabolism and hormone signal transduction may play a role in the same biological process or at least have a close functional connection.

**Figure 5:**
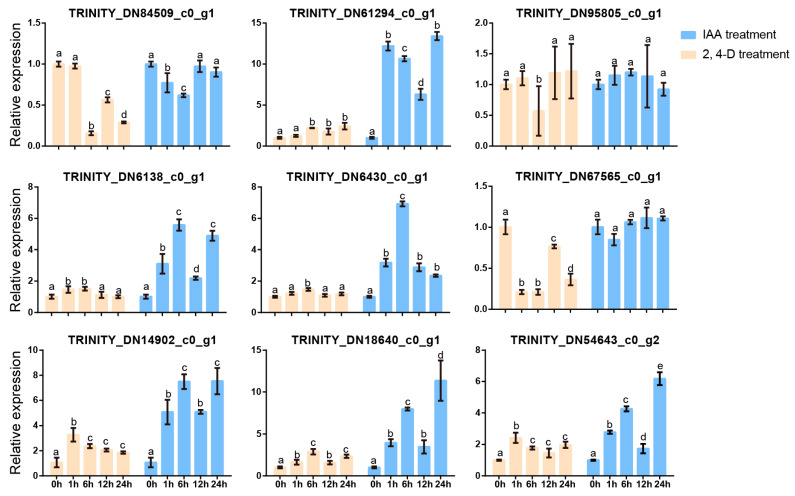
Expression analysis of nine auxin-related genes at different time points after IAA and 2,4-D treatment. Lowercase letters indicate significant differences. Differential analysis was performed by SPSS 22.0 software using the LSD method with *p* < 0.05.

**Figure 6:**
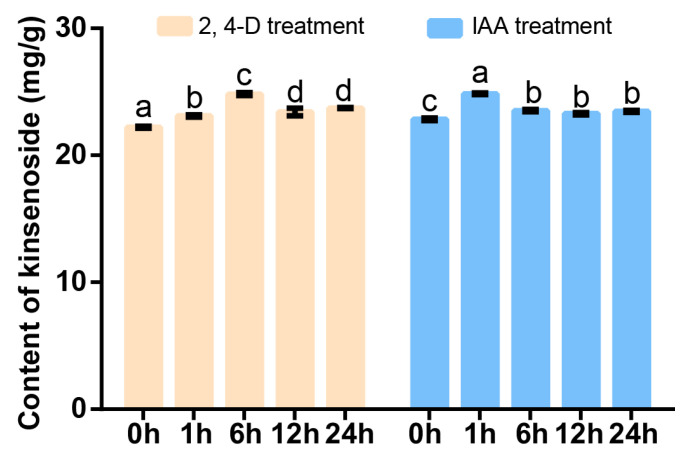
Kinsenoside content analysis of *A. roxburghii* treated with IAA and 2,4-D at different time points. Letters indicate significant difference. Lowercase letters indicate significant differences. Differential analysis was performed by SPSS 22.0 software using the LSD method with *p* < 0.05.

We further annotated and analyzed the expression profiles of genes related to hormones. The results showed that the hormone-related KEGG pathway contains a total of sixteen genes, nine of which are related to auxin. Therefore, we selected *A. roxburghii* cv. ‘Jian ye’, which had the highest kinsenoside content of the three species of *A. roxburghii,* added exogenous auxin-like substances (IAA and 2,4-D), and detected expression changes in the nine genes through RT-qPCR (Supplementary Materials, Table S1). The results indicated that *TRINITYDN95805_c0ug1* showed little response to both hormone treatments. *TRINITYDN6430_c0ug1* and *TRINITYDN67565_c0ug1* responded to IAA and 2,4-D, respectively. The remaining genes responded to IAA and 2,4-D, and their expression levels significantly increased after hormone treatment (Figure 5). Therefore, we further detected the kinsenoside content in *A. roxburghii* after IAA and 2,4-D treatments, and our results indicate that auxin promotes the synthesis of kinsenoside (Figure 6).

### 3.4. Identification of the UDP Glycosyltransferase Gene Family of A. roxburghii

**Figure 7:**
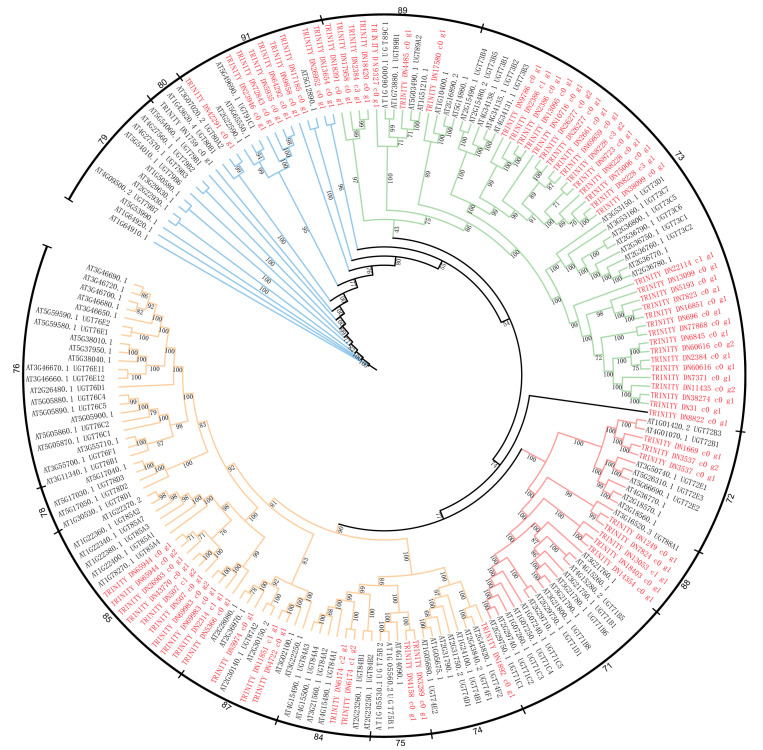
Phylogenetic tree of UDP glycosyltransferase gene family members from *A. roxburghii* and *A. thaliana.* The red and black gene IDs represent UDP glycosyltransferase gene family members in *A. roxburghii* and *A. thaliana*, respectively. We use blue, green, red, and orange evolutionary clades to represent different groups of UDP glycosyltransferase gene families.

At present, the biosynthetic pathway of kinsenoside is not clear; however, another important synthase, UDP glycosyltransferase, is necessary for kinsenoside biosynthesis. Therefore, in order to understand the evolutionary relationship of the UDP glycosyltransferase gene family in *A. roxburghii*, this study identified 73 UDP glycosyltransferase family members in *A. roxburghii* transcripts and constructed a phylogenetic tree based on 113 members from *A. thaliana*. The results show that the phylogenetic tree can be divided into four main clades as a whole (Figure 7). Furthermore, we divided all members into 15 subfamilies, namely 79, 80, and 91 in the blue main clade, 73 and 89 in the green main clade, 71, 72, and 88 in the red main clade, and 74, 75, 76, 78, 84, 85, and 87 in the orange main clade [25]. Each subfamily in the UDP glycosyltransferase family has a substrate that is specifically recognized, such as subfamilies 79 and 91, which use flavonoids, saponins, and cytokinins as substrates. From the perspective of the entire phylogenetic tree, the UDP glycosyltransferase family from *A. roxburghii* is distributed in almost every subfamily, indicating its functional conservation.

### 3.5. Analysis of Expression Patterns of UDP Glycosyltransferase Gene Family Members in Three Species of A. roxburghii

**Figure 8:**
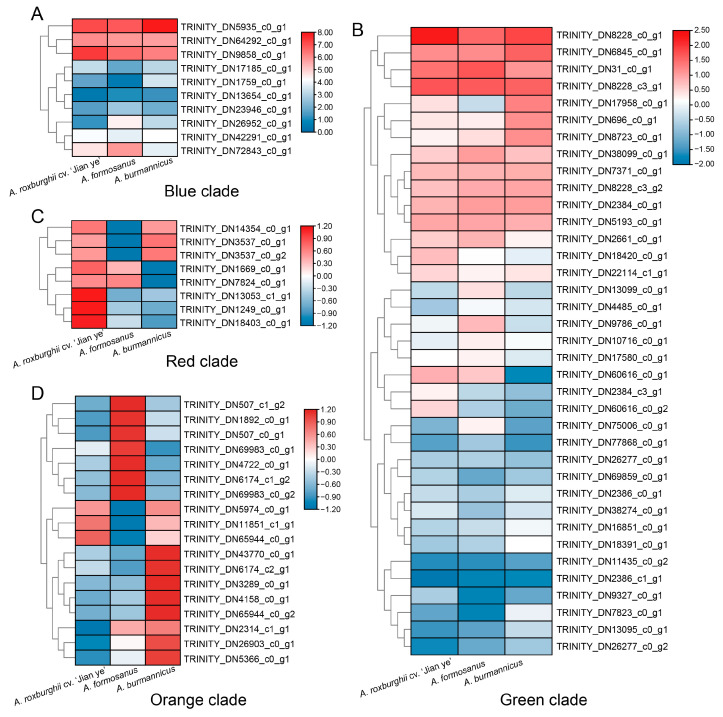
Expression profiles of UDP glycosyltransferase family members in different branches of *A. roxburghii*. (**A**–**D**) correspond to the branches in the phylogenetic tree of Figure 7, respectively.

To understand the expression pattern of UDP glycosyltransferase family members in three species of *A. roxburghii*, we first classified all genes based on the phylogenetic tree. In the blue and green clades, the high and low expression of UDP glycosyltransferase members in the three species of *A. roxburghii* are essentially the same, indicating that the members of these two clades may not be the key factors affecting kinsenoside synthesis (Figure 8A,B). In the red clade, all eight members of the UDP glycosyltransferase family are highly expressed in *A. roxburghii* cv. ‘Jian ye’; meanwhile, three members are highly expressed in *A. burmannicus*, and two members are highly expressed in *A. formosanus* (Figure 8C). Therefore, we believe that members of the UDP glycosyltransferase family in the red main clade may be key genes promoting kinsenoside synthesis. Finally, the UDP glycosyltransferase family members in the orange main clade are highly expressed in *A. formosanus* and *A. burmannicus*, indicating that they may not have a significant impact on kinsenoside synthesis (Figure 8D).

**Figure 9:**
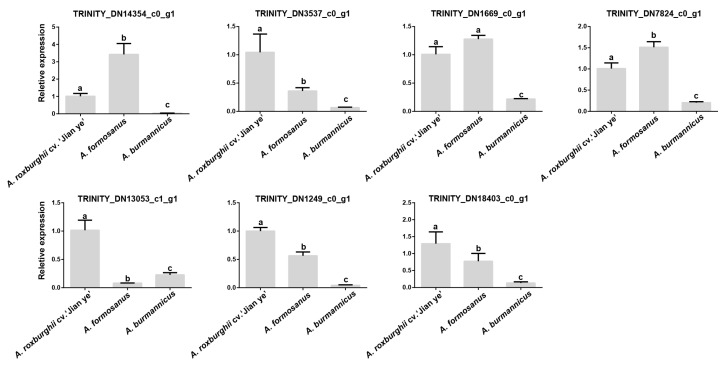
Relative expression analysis of seven genes in the red clade. Lowercase letters indicate significant differences. Differential analysis was performed by SPSS 22.0 software using the LSD method with *p* < 0.05.

To understand the role of seven genes in the red main clade in kinsenoside synthesis, we further investigated the expression patterns of the seven genes in the three species of *A. roxburghii* (Figure 9). The results showed that within the expression levels of seven genes among the three varieties of *A. roxburghii*, four genes (*TRINITYDN3537_c0ug1, TRINITYDN13053_c1_g1, TRINITYDN1249_c0ug1,* and *TRINITYDN18403_c0ug1*) were significantly expressed in *A. roxburghii* cv. ‘Jian ye’. In addition, *TRINITYDN7824_c0ug1* and *TRINITYDN1669_c0ug1* were highly expressed in both *A. roxburghii* cv. ‘Jian ye’ and *A. formosanus*. The above results indicate that the four genes with high specific expression levels in *A. roxburghii* cv. ‘Jian ye’ may be key UDP glycosyltransferases involved in kinsenoside synthesis in this species.

**Figure 10:**
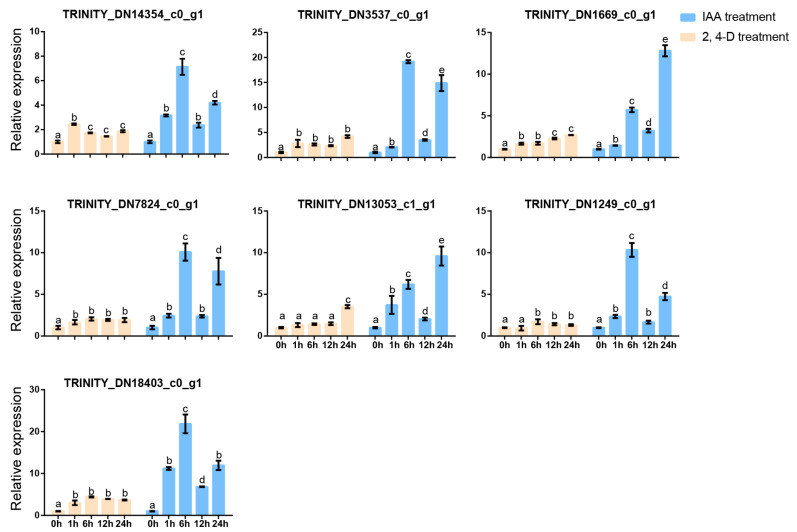
Expression analysis of seven genes in the red clade at different time points after IAA and 2,4-D treatment. Lowercase letters indicate significant differences. Differential analysis was performed by SPSS 22.0 software using the LSD method with *p* < 0.05.

The above findings suggest that auxin and UDP glycosyltransferase may be involved in the synthesis of kinsenoside in *A. roxburghii*. Therefore, we further investigated the expression of the above-mentioned glycosyltransferase genes after auxin treatment. The results showed that all UDP glycosyltransferases in the red clade could respond to auxin regulation, and their expression levels significantly increased after auxin treatment (Figure 10). Meanwhile, we determined the kinsenoside content after exogenous auxin treatment using the HPLC method. The results showed that after treatment with IAA and 2,4-D, the kinsenoside content significantly increased (Figure 6). Therefore, we believe that auxin may promote the synthesis of kinsenoside by regulating auxin-related genes or related glycosyltransferases.

## 4. Discussion

By using transcriptome sequencing technology to compare gene expression trends in plants in different environments or tissues from different locations and combining transcriptome data with phenotype data, the key genes regulating plant traits can be quickly and effectively identified [26]. As a precious medicinal herb, *A. roxburghii* has significant value in the medical and healthcare fields. However, high-throughput sequencing research on *A. roxburghii* began relatively late, and there are very few related studies, which only report its heat stress [5], flavonoids [6,8,9], and pigment synthesis [7]. As kinsenoside is the most important active ingredient in *A. roxburghii*, we determined the kinsenoside content in whole plants belonging to three species of *A. roxburghii* collected from inland China and Taiwan Province. We found that the kinsenoside content in *A. roxburghii* cv. ‘Jian ye’ was significantly higher than in *A. formosanus* and *A. burmannicus.* Based on this, we study used transcriptome sequencing and metabolomics analysis to find that *A. roxburghii* cv. ‘Jian ye’ has a large number of DEGs and accumulated metabolites compared to the *A. formosanus* and *A. burmannicus* species. The results showed that a large number of genes and metabolites in *A. roxburghii* cv. ‘Jian ye’ were enriched via the metabolism of vitamins, cofactors, amino acids, lipids, and other substances. Therefore, this study suggests that gene and metabolite differences in *A. roxburghii* cv. ‘Jian ye’ may contribute to its accumulation of nutrients and medicinal components.

Due to the unknown synthetic route of kinsenoside, we are unable to further extract its key genes. Therefore, we constructed a correlation network, which is widely used in the search for key genes in horticultural crops [27,28,29,30]. We used the WGCNA method to integrate the transcriptome data of *A. roxburghii* with the content data of kinsenoside and identified genes that may be involved in kinsenoside synthesis. The results showed that the black and green-yellow modules were highly correlated with the content of kinsenoside. In addition, the genes of these two modules are extensively enriched in glucose metabolism and auxin signaling transduction. Therefore, we speculate that there is a certain correlation between the synthesis of kinsenoside and auxin. However, genes cannot directly mediate the synthesis of kinsenoside. Therefore, there must be a pathway in nature that synthesizes kinsenoside through the glycosylation of 3HBL and six-carbon glucose, making glycosyltransferase a candidate gene for this process [12,13]. We identified all members of the UDP glycosyltransferase gene family in the transcriptome of *A. roxburghii* and constructed a phylogenetic tree with members from *Arabidopsis*. The results showed that the UDP glycosyltransferase gene family members of *A. roxburghii* clustered with *Arabidopsis* into four main branches, which is consistent with previous research results [31,32]. However, due to the lack of publicly available genomic data for *A. roxburghii*, the number of UDP glycosyltransferase gene family members in *A. roxburghii* is relatively small, with only 73. We characterized the expression data of all UDP glycosyltransferase members in *A. roxburghii*, and the results showed that in high-kinsenoside species, the red-branched UDP glycosyltransferase was highly expressed in *A. roxburghii* cv. ‘Jian ye’, and our qPCR results also confirmed this. The UDP glycosyltransferase members in the red branch of *A. roxburghii* are UDP glycosyltransferase 72 and UDP glycosyltransferase 88, belonging to group E. The glycosyltransferases in group E, as the largest expansion group in angiosperms, account for 20–25% of the entire UDP glycosyltransferase gene family [25]. In previous studies, the main functions of UDP glycosyltransferase 72 were shown to be lignin synthesis and cell wall lignification [33]. In addition, UDP glycosyltransferase 88 has been shown in previous studies to catalyze the glycosylation of substrates at the 4-O and 7-O positions [34,35,36,37,38]. However, the glycosylation site of 3HBL is 3-O, so we speculate that UDP glycosyltransferase in group E may still have unknown functions. In addition, to further validate the relationship between auxin, auxin-related genes, UGP glycosyltransferase, and kinsenoside, we further treated *A. roxburghii* cv. ‘Jian ye’ with IAA and 2,4-D. The results showed that with exogenous hormone treatment, auxin-related genes, UGP glycosyltransferase, and kinsenoside were significantly upregulated. Therefore, we speculate that the synthesis of kinsenoside is significantly correlated with auxin signaling and UDP glycosyltransferase.

In summary, using transcriptomics and metabolomics sequencing techniques, we found that *A. roxburghii* cv. ‘Jian ye’ is richer in nutritional and medicinal components than the two other species investigated. In addition, through transcriptome data characterization and qPRC analysis, we speculate that UDP glycosyltransferase 72 and UDP glycosyltransferase 88 in group E may be involved in the synthesis of kinsenoside. Further synthetic biology evidence is needed for these results, but our research also provides an important bioinformatics foundation for the study of kinsenoside synthesis.

## Data Availability

All sequencing data, including those of the genome and transcriptome, can be found in the China National Center for Bioinformation database (PRJCA014460).

## Supplementary Materials

The following supporting information can be downloaded at https://www.mdpi.com/article/10.3390/plants14050688/s1: Table S1: Primers used in this study; Table S2: Statistical table of the sequencing data; Figure S1: Determination of kinsenoside in three species of *A. roxburghii;* Figure S2: Analysis of DEGs in transcriptome data of three species of *A. roxburghii*; Figure S3: OPLS-DA method analysis; Figure S4: Differential cumulative metabolite analysis of three species of *A. roxburghii;* Figure S5: Weighted gene co-expression network analysis clustering of gene modules related to kinsenoside; Figure S6: Transcriptome FPKM analysis of genes related to auxin.

## Abbreviations

*A. roxburghii*	*Anoectochilus roxburghii*
3HBL	3-hydroxy-γ-butyrolactone
DEGs	differentially expressed genes
RT-qPCR	real-time fluorescence quantitative PCR
LSD	least significant difference
PCA	principal component analysis
PCA 1	principal component analysis 1
PCA 2	principal component analysis 2
VIP	Variable Importance in Projection
KEGG	Kyoto Encyclopedia of Genes and Genomes
WGCNA	weighted gene co-expression network analysis
FPKM	fragments per kilobase of exon model per million mapped fragments
Q 30	Qphred 30
