# Supplementary material for: Transcriptomic and Metabolomic Insights into Key Genes Involved in Kinsenoside Biosynthesis in Anoectochilus roxburghii
Source: Plants (Basel). 2025 Feb 24;14(5):688. doi: 10.3390/plants14050688 (PMC11902215; doi:10.3390/plants14050688)
Supplement: Supplementary file 1 [file plants-14-00688-s001.zip › Supplementary figure.pdf]

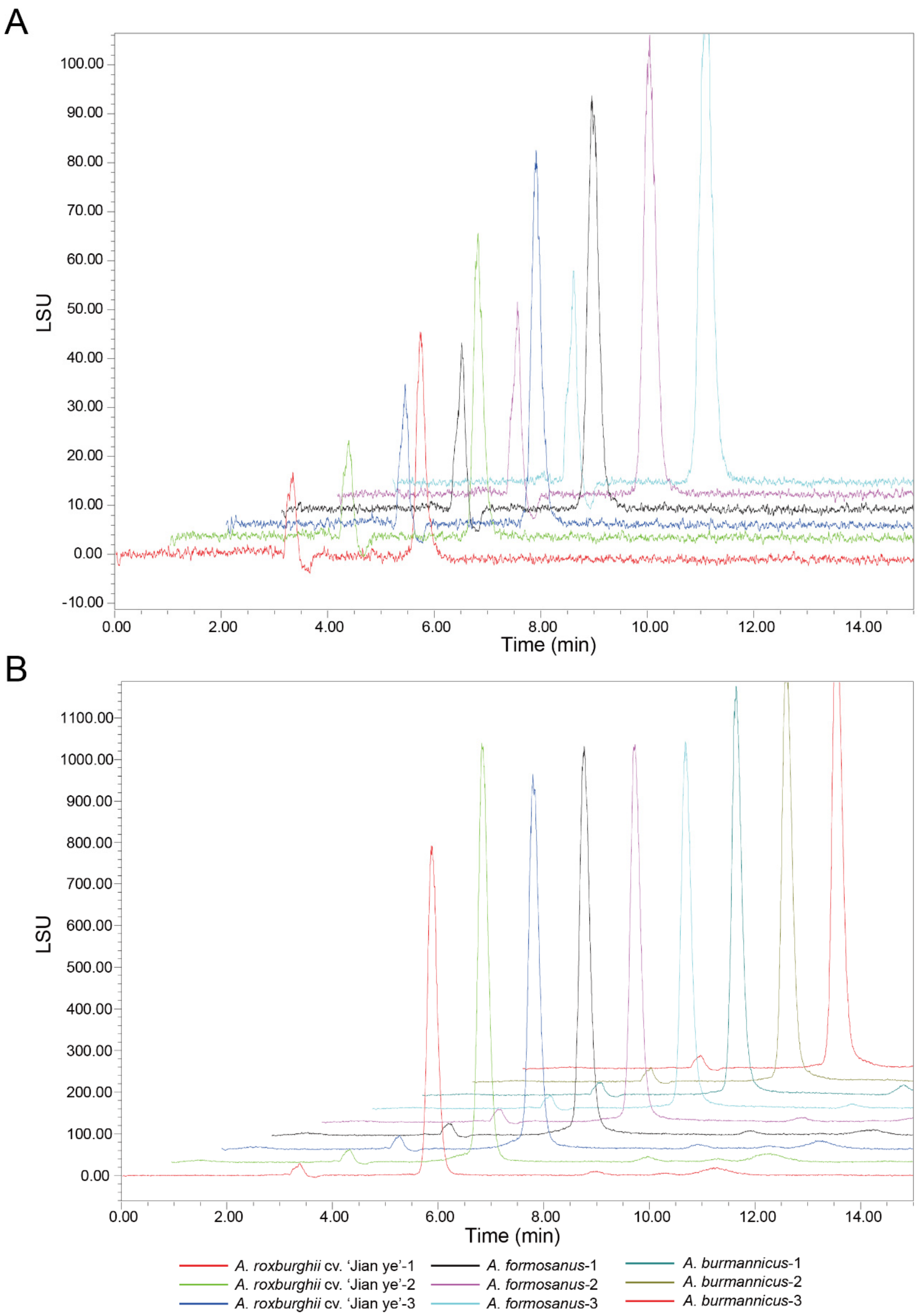

Figure S1. Determination of kinsenoside in three species of *A. roxburghii.* A, injections of 1.5, 2.0, 2.5, 3.0, 3.5, and 4.0, B, Chromatographic peak diagrams for the determination of kinsenoside in three species of *A. roxburghii*, n=3.

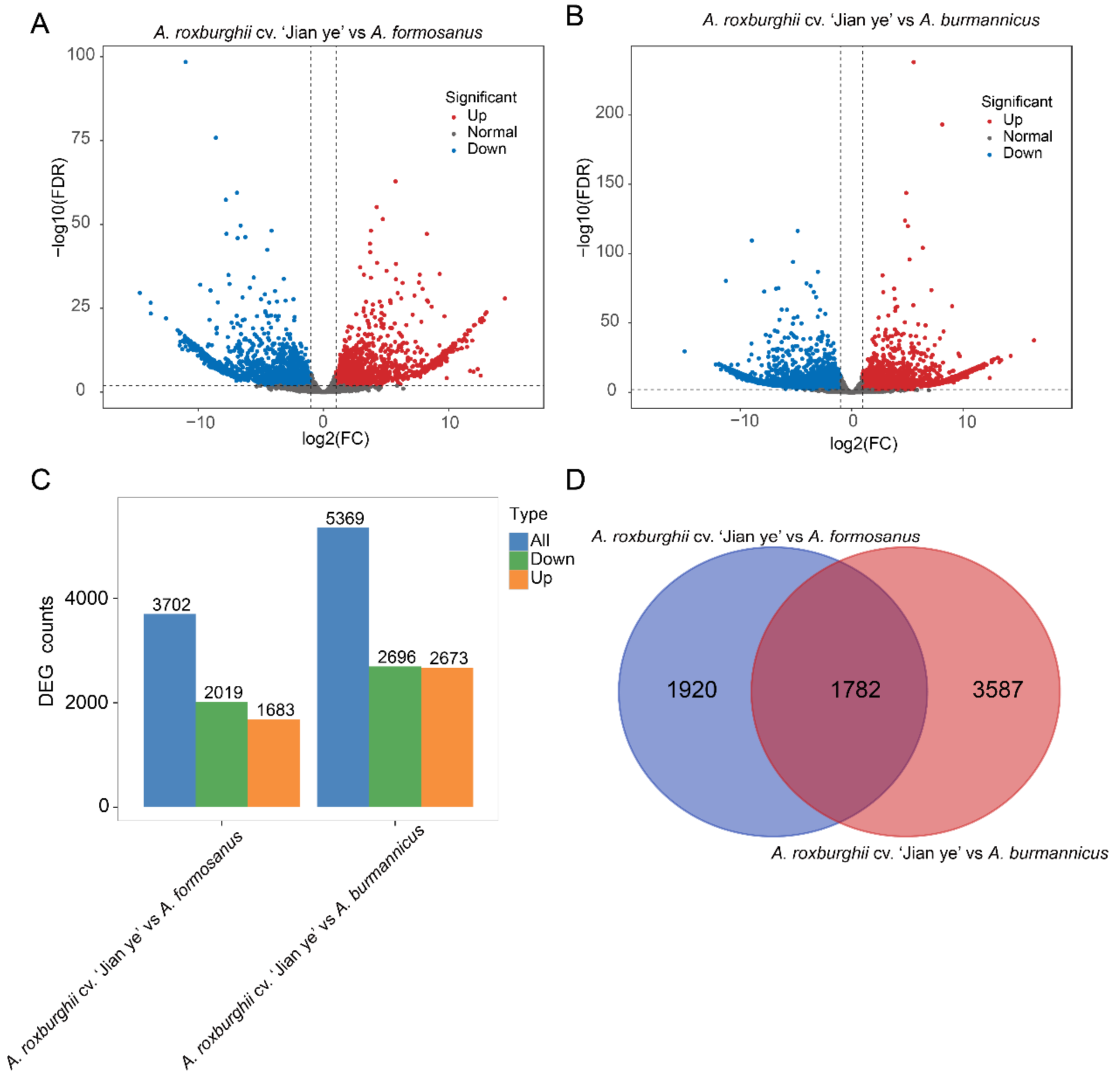

Figure S2. Analysis of DEGs in transcriptome data of three species of *A. roxburghii*. A， B: Volcanic map of DEGs, C: Histogram of the number of DEGs, D: DEGs Venn diagram.

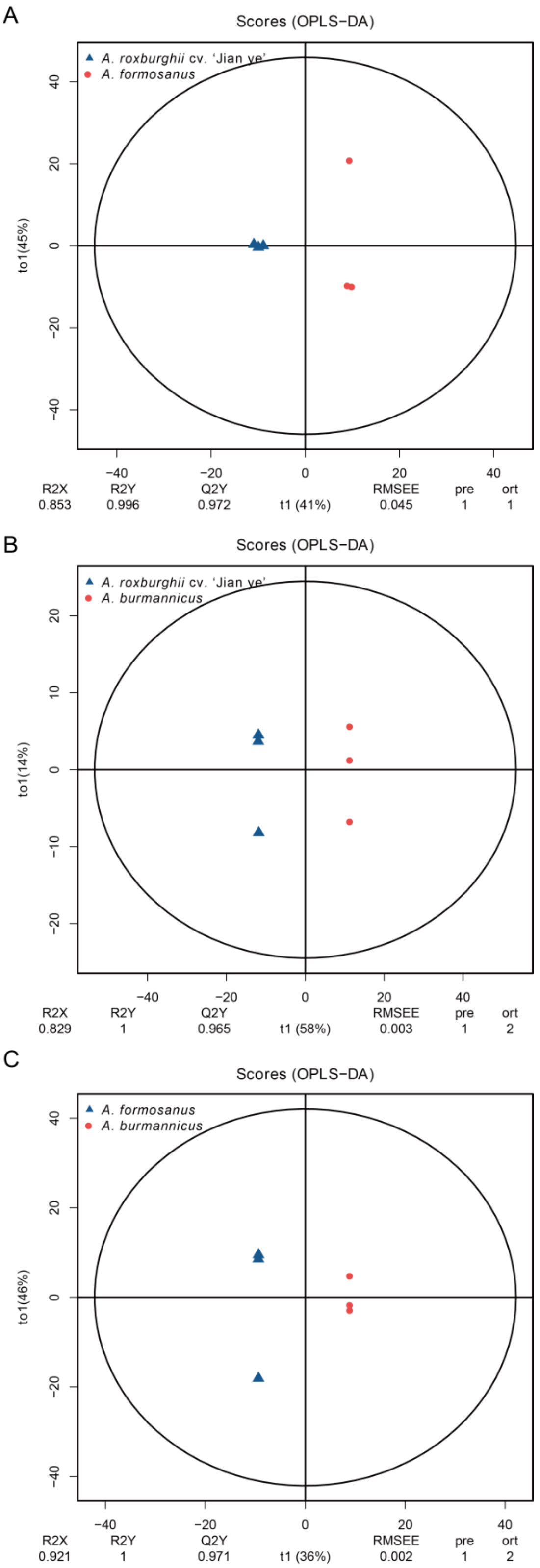

Figure S3. OPLS-DA score chart. The x-axis (t1) represents the predictive component (between-group difference component), and the y-axis (to1) denotes the orthogonal component (within-group difference component). The percentage on the horizontal y-axis indicates the proportion of the total variance explained by the respective component. Below the plot, the model parameters are annotated, including R2X, R2Y, Q2Y, RMSEE (root mean square error of estimation), pre (number of predictive components), and ort (number of orthogonal components).

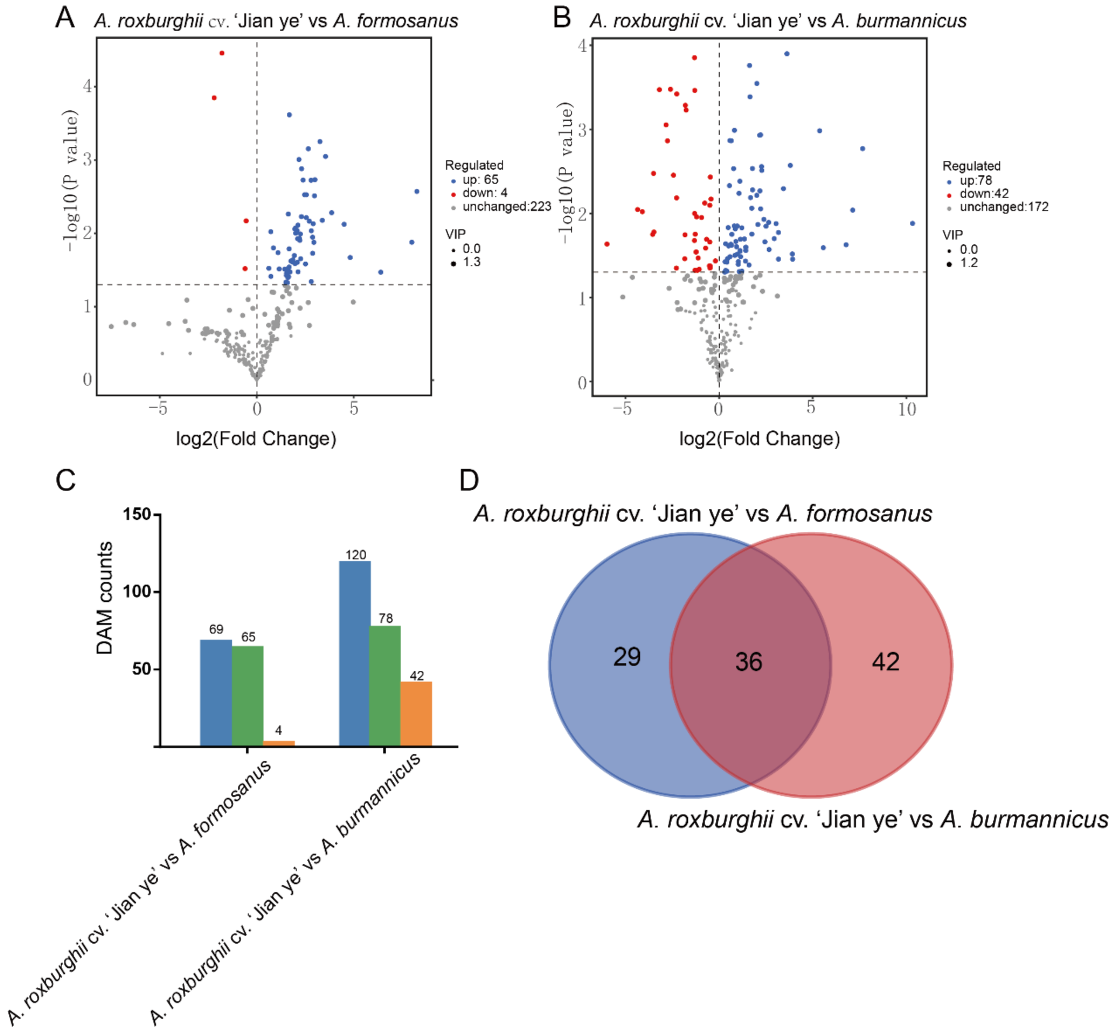

Figure S4. Differential Cumulative Metabolite Analysis of Three species of *A. roxburghii*. A，B: Volcanic map of differential cumulative metabolites, C: Statistical histogram of accumulated metabolite quantity differences, D: Differential accumulation metabolite Venn diagram.

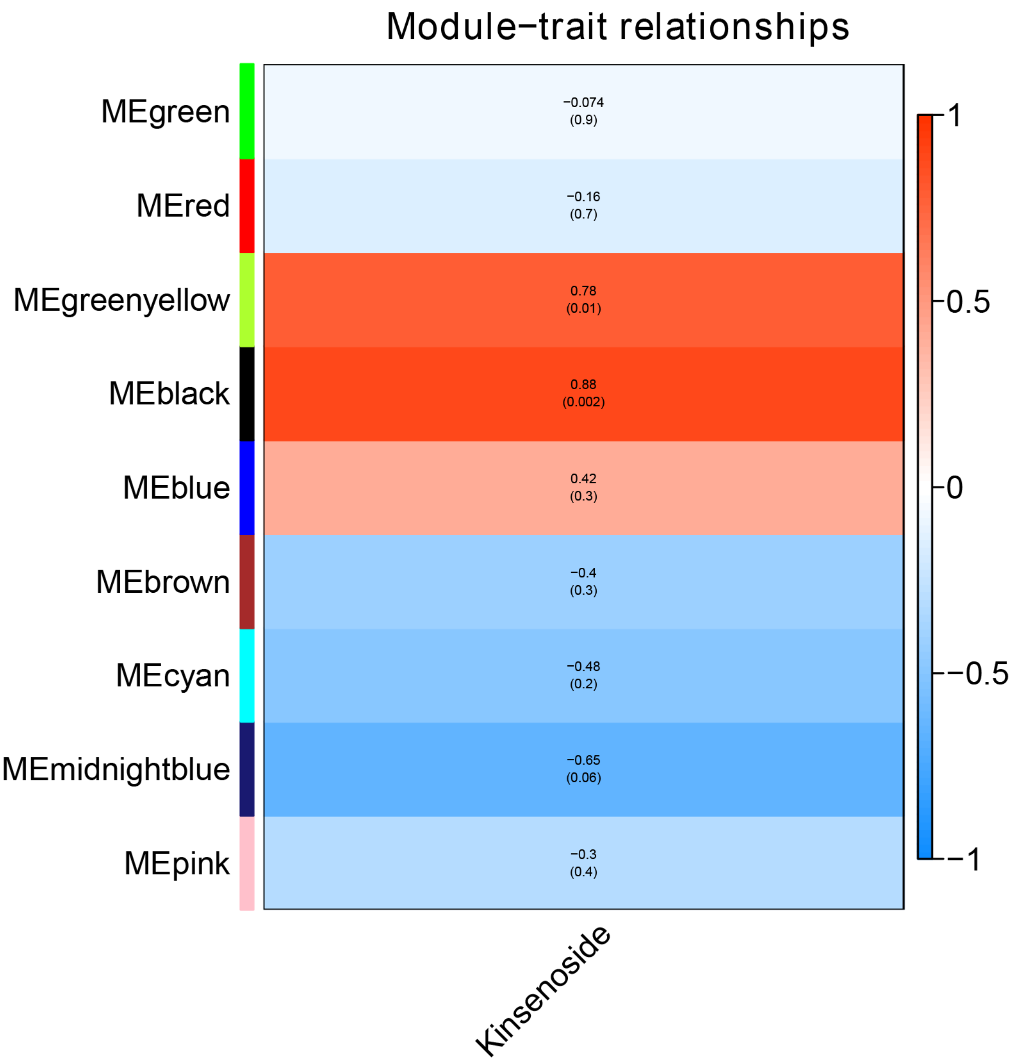

Figure S5. Weighted gene co expression network analysis clustering of gene modules related to kinsenoside.

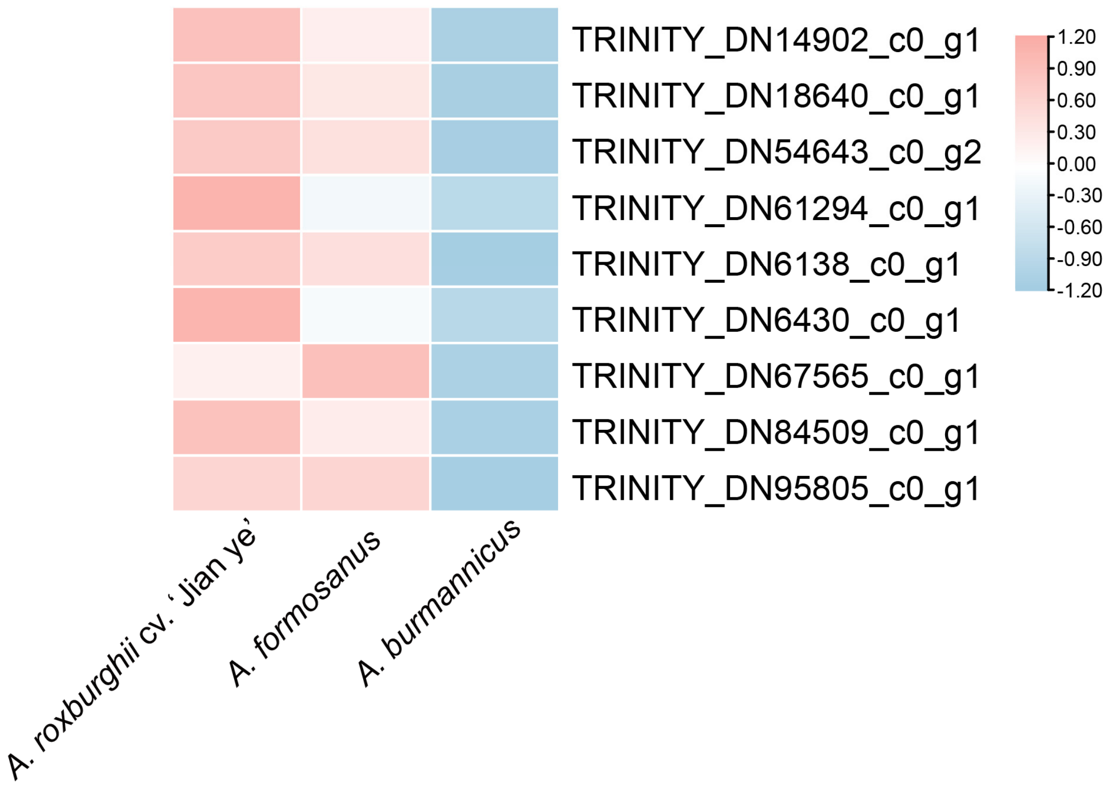

Figure S6. Transcriptome FPKM analysis of genes related to auxin.
